# Multidrug stewardship and adherence to guidelines in >200,000 direct-to-consumer Telemedicine encounters

**DOI:** 10.31744/einstein_journal/2024AO0707

**Published:** 2024-06-21

**Authors:** Flavio Tocci Moreira, Tarso Augusto Duenhas Accorsi, Karine De Amicis, Karen Francine Köhler, Renata Albaladejo Morbeck, Eduardo Cordioli, Carlos Henrique Sartorato Pedrotti

**Affiliations:** 1 Hospital Israelita Albert Einstein Telemedicine Department São Paulo SP Brazil Telemedicine Department, Hospital Israelita Albert Einstein, São Paulo, SP, Brazil.

**Keywords:** COVID-19, Guideline adherence, Telemedicine, Prescriptions, Drug prescriptions, Referral and consultation, Communicable diseases, Patient discharge, Quality indicators, health care, Respiratory tract infections, Coronavirus Infections

## Abstract

**Objective:**

The quality of care and safety for Telemedicine-discharged patients with suspected respiratory infections are closely related to low rates of prescriptions of unjustified and high-risk medications. This retrospective study aimed to assess adherence to the current COVID-19 guidelines in direct-to-consumer telemedicine encounters at a large center using multidrug stewardship protocols.

**Methods:**

A quarterly electronic survey utilizing medical records of individual physician care assessed various quality indicators. Physicians received ongoing adaptive feedback based on personal metrics, with Telemedicine Center recommendations derived from the 2020 Infectious Diseases Society of America guidelines. The study included all consecutive adults with new respiratory symptoms in the last 14 days who sought spontaneous Telemedicine consultations between March 2020 and August 2021. This study analyzed patients with suspected or confirmed COVID-19 and other airway infections.

**Results:**

Of the 221,128 evaluated patients, 42,042 (19%) had confirmed COVID-19; 104,021 (47%) were suspected to have COVID-19; and, 75,065 (33%) had other diagnoses. Patients with suspected or confirmed COVID-19 had a mean (+DP) age of 35±12 years. A total of 125,107 (85.65%) patients were managed at home, 2,552 (1.74%) were referred for non-urgent in-office reassessment, and 17,185 (11.7%) were referred to the emergency department for whom there was no further treatment recommendation. The antibiotic rate in confirmed or suspected COVID-19 cases was 0.46%/0.65% and that for non-evidence-based prescriptions was 0.01%/0.005%.

**Conclusion:**

Guideline training and Telemedicine consultation feedback may lead to lower antibiotic and antimicrobial prescriptions in suspected and confirmed COVID-19 cases. Multidrug stewardship protocols may improve guideline adherence and reinforce the quality of care and safety in Telemedicine encounters.

## In Brief

Moreira et al. analyzed 221,129 telemedicine encounters and revealed that guideline-based training and personalized feedback for physicians substantially reduced antibiotic and antimicrobial prescriptions in suspected and confirmed COVID-19 cases, highlighting the effectiveness of multidrug stewardship protocols.

## Highlights

▪ Up to 75% of COVID-19 telemedicine patients receive unnecessary drugs; this highlights the need for research.

▪ This study assessed the effect of guideline training and feedback on 200,000 telemedicine visits in Brazil.

▪ Under 2% of patients with COVID-19 received non-guideline recommended antimicrobials, reflecting high adherence rates.

▪ Targeted interventions significantly enhance guideline compliance for safer telemedicine prescriptions.

## Graphical abstract

**Figure:**
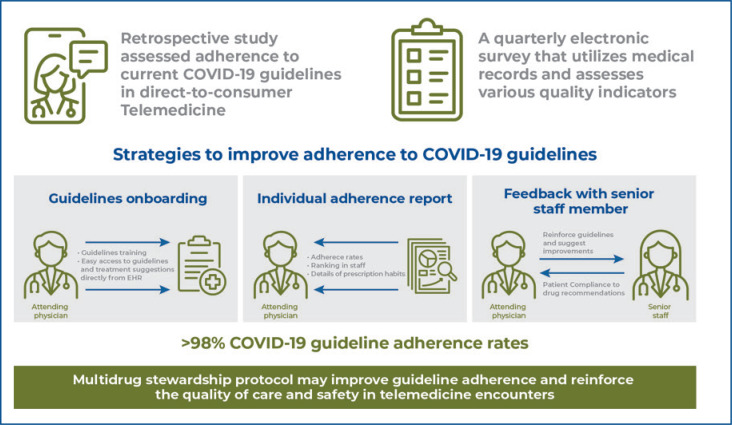

## INTRODUCTION

Due to the coronavirus disease (COVID-19) pandemic, patients routinely use Telemedicine (TM) for their first contact with the healthcare system.^(1)^ Providers should identify conditions that can be managed remotely and immediately refer at-risk patients in a constant, repetitive, and, often, tiresome routine. The quality of care and safety in TM-discharged patients are closely related to the low rates of prescriptions of unjustified antimicrobial and high-risk medications.^(2)^ Although decision support algorithms are available, adequate adherence to these recommendations during direct-to-consumer TM evaluations is poorly understood.

## OBJECTIVE

This study aimed to analyze adherence to the current COVID-19 guidelines in Telemedicine encounters at a large center adopting multidrug stewardship protocols.

## METHODS

This unicentric study with an observational and retrospective design enrolled patients from March 2020 to August 2021. This study was performed at the Telemedicine Center of *Hospital Israelita Albert Einstein* (São Paulo, Brazil), a general hospital where board-certified physicians perform all TM evaluations. The study and consent waivers (data were analyzed anonymously) were approved by the *Hospital Israelita Albert Einstein* Review Board (CAAE: 51426221.3.0000.0071; #5.032.779). De-identified datasets, including age, encounter diagnosis (*International Classification of Diseases, Tenth Revision* [ICD-10] codes), and treatment plans, were used in this analysis.

Multidrug stewardship protocols were implemented according to the current international guidelines. In April 2020, the Infectious Diseases Society of America (IDSA) Guidelines on the Treatment and Management of Patients with COVID-19 were published,^(3)^ which supported the update of the institutional guidelines adopted almost all the time of the study.^(4)^ The IDSA guidelines recommend against the liberal prescription of oral corticosteroids, antibiotics, chloroquine, and hydroxychloroquine in hospitalized patients with COVID-19. At that time, there was no mention of ivermectin and nitazoxanide, which were also not included in the updated guidelines by this society and were considered non-routine recommendations. Recommendations meant for hospitalized patients were extrapolated to low-risk patients treated in outpatient settings. During the study, it was considered good practice only to prescribe symptomatic drugs (analgesics, antihistamines, antitussives, and nasal hygiene) as needed in patients managed remotely.

Our study conducted at the Telemedicine Center followed strict safety measures and considered the virtual management of high-risk patients to be unsafe; therefore, high-risk patients were not treated and oriented in the remote encounter. Severe viral infections cannot be differentiated from bacterial infections accurately through virtual care. However, regardless of the etiology of the condition, red flags indicating severity imply immediate referral for in-person emergency care. The emergency department referral criteria are shown in table 1. The study population included all consecutive adult patients with any new respiratory symptoms in the last fourteen days who sought TM consultations spontaneously, and all patients with a suspected (J06.9) or confirmed (U07.1 and B34.2) COVID-19 diagnosis and other diagnoses of airway infections were included in the analysis. Patients who were asymptomatic or diagnosed with other conditions were excluded. Government newsletters on the COVID-19 pandemic have been periodically released. According to government data, the prevalence of positive RT-PCR-based COVID-19 test results in the study period was 37.25%±10.01%. The Telemedicine Center manager conducted a quarterly electronic survey based on the medical records of several indicators related to care among all physicians in the service. The following general indicators were measured: number of queries performed, number of hours of work, NPS, resoluteness, percentage of medical certificates, rate of prescription of antibiotics, average number of days - medical certificate, median duration (min) appointment, returns, pediatric appointments, and percentage of work between 00:00 a.m. and 06:00 p.m. The quality indicators measured included medical history in less than 100 characters, 14-day certificates and complaints with less than 1 day, on-protocol controlled drugs, complementary examination requests outside the institutional protocol, prescription of ophthalmological corticosteroids, use of antibiotics for acute gastroenteritis, prescription of homeopathy/multivitamins, divergent medical orientation, error in antibiotic form, risk of cross-allergy in NSAIDs, unapproved prescription drugs, incomplete physical examination, and prescription of drugs not approved for COVID-19. Immediately after each personal data collection, the medical chief of the Telemedicine Center provided individualized feedback to each physician and highlighted points for improvement to adapt to the current guidelines. They were provided study material, and the guidelines were easily accessible during remote encounters.

**Table 1:** Criteria for referral to the emergency room

Age		
	Children	Under 5 years old
	Older people	Above 60 years old
Chronic diseases		
	Respiratory	Asthma
		Chronic Obstructive Pulmonary Disease
		Interstitial diseases
	Cardiovascular	Heart failure
	Kidneys	Chronic kidney dysfunction
	Liver	Cirrhosis
	Hematologic	Sickle cell anemia
	Neurological	Epilepsy
		Previous stroke
	Low immunity	Medicine-induced
		Chemotherapy
		HIV/AIDS
Pregnancy		
	Pregnant	Any period
	Postpartum	Up to 2 weeks
Nursing home residents		
	Any age	Any length-of-stay
Warning clinical signs		
	Worsening of previous condition	Doble worsening
	Suggestive of bacterial infection	Increased cough
		Sputum
		Chest pain
		Chest pain
		Hypotension
		Fever relapse
	Suggestive of severe viral infection	Persistence of high fever
		Dyspnea
		Cyanosis
	Rare conditions		
	Myositis	Any degree
	Guillain–Barré syndrome	Any suspicion

## RESULTS

Of the 221,128 evaluated patients, 42,042 (19%) had confirmed COVID-19; 104,021 (47%) were suspected of having COVID-19; and 75,065 (33%) had other diagnoses. The final diagnostic hypotheses for non-COVID-19-related cases are presented in table 2. Patients with suspected or confirmed COVID-19 had a mean (+DP) age of 35±12 years. A total of 125,107 (85.65%) patients were managed at home, 2,552 (1.74%) were referred for non-urgent in-office reassessment, and 17,185 (11.7%) were referred to the emergency department, for whom there was no further treatment recommendation. The leading causes for urgent referrals are listed in table 3.

**Table 2:** Final diagnosis hypotheses of non-COVID-19 cases

ICD-10	Description	n (%)
J06	Acute upper respiratory infections of multiple and unspecified sites	14,610 (19.4)
Z20	Contact with and (suspected) exposure to communicable diseases	10,242 (13.6)
J01	Acute sinusitis	4,464 (5.9)
J03	Acute tonsillitis	4,382 (5.8)
J00	Acute nasopharyngitis [common cold]	4,359 (5.8)
R50.9	Fever, unspecified	4,219 (5.6)
R05	Cough	3,697 (4.9)
B34.9	Viral infection, unspecified	3,681 (4.9)
B34	Viral infection of unspecified site	3,367 (4.4)
J02	Acute pharyngitis	3,249 (4.3)
R06.0	Dyspnea	3,024 (4.0)
Z20.8	Contact with and (suspected) exposure to other communicable diseases	2,415 (3.2)
J30	Vasomotor and allergic rhinitis	1,654 (2.2)
J30.8	Other allergic rhinitis	1,490 (1.9)
R07.0	Pain in the throat	1,176 (1.5)
J11	Influenza due to unidentified influenza virus	1,113 (1.4)
R50	Fever of other and unknown origin	1,030 (1.3)
Z20.9	Contact with and (suspected) exposure to unspecified communicable disease	745 (0.9)
J03.9	Acute tonsillitis, unspecified	681 (0.9)

**Table 3:** Leading causes for emergency department referral

Symptom	n (%)
Persistent fever	3,318 (19.3)
Not specified	2,619 (15.2)
Chest pain	2,177 (12.6)
Chest pain and dyspnea	2,108 (12.2)
Other reasons	1,737 (10.1)
Persistent cough	941 (5.4)
Persistent symptoms	593 (3.4)
No clear red flag	473 (2.7)
Severe headache	371 (2.1)
Desaturation	234 (1.3)
Severe tonsilitis	227 (1.3)
Intense prostration	226 (1.3)
Bronchospasm	194 (1.3)
Risk group	170 (0.9)
Clinical deterioration	167 (0.9)
Dengue suspicious	101 (0.5)
Earache	90 (0.5)

Of all the included patients managed at home, 107,772 (86,22%) received a pharmacological treatment plan. Table 4 describes the prevalence of drug class recommendations according to the main diagnosis. A total of 17,245 patients (13.78%) did not receive drug recommendation.

**Table 4:** Medicines prescribed according to diagnosis

	Confirmed COVID-19 ICD: U07.1/B34.2 n (%)	Suspected COVID-19 ICD: J06.9 n (%)
Analgesics	20,773 (49.41)	78,407 (75.37)
Antihistamines	6,412 (15.25)	24,467 (23.52)
Antitussives	2,495 (5.93)	6,177 (5.94)
Nasal saline	11,642 (27.69)	50.117 (48.18)
Oral corticosteroids	513 (1.22)	1,339 (1.29)
Antibiotics	193 (0.46)	672 (0.65)
Other drugs*	6 (0.01)	5 (0.005)

* Chloroquine, hydroxychloroquine, ivermectin, nitazoxanides.

ICD: International Code Disease.

## DISCUSSION

Medical TM evaluations of patients with acute respiratory disease during the COVID-19 pandemic led to a reduction of at least three-quarters of immediate face-to-face assessments in the emergency department.^(5)^ Although the volume of TM encounters has exponentially increased worldwide because of the pandemic, data regarding clinical quality standards and guideline adherence are still scarce in this setting. The purpose of clinical practice guidelines is to synthesize the best available evidence to support clinical decision-making, which improves the quality of care and patient outcomes and provides the most cost-effective care. However, the publication of guidelines does not guarantee guideline implementation or clinician adherence. Therefore, adequate stewardship during protocol implementation is imperative.^(6)^ A previous study by the same group found high levels of adherence to antibiotic prescriptions with stewardship protocols in TM encounters before the pandemic.^(7)^ Importantly, unnecessary antibiotic use is likely high in patients with COVID-19. A meta-analysis showed that as many as three-quarters of patients with COVID-19 received antibiotics, and the prescription rate was significantly higher than the estimated prevalence of bacterial coinfection.^(8)^ In a previous study based on a specialized TM assessment of inpatients using antibiotics, 67% of prescriptions were for respiratory infections, and 55% prescriptions were considered inappropriate.^(9)^

However, a meta-analysis of 14 studies on antibiotic stewardship protocols has proved its benefits in increasing adherence to guidelines and reducing prescription rates without significantly affecting patient clinical outcomes.^(10)^ Therefore, TM provides a tool for decision support, which can promote better antibiotic selection and reduce bacterial resistance.^(11)^ An analysis of 6 studies found a significant reduction in errors in the postimplementation of antiretroviral stewardship.^(12)^ Furthermore, a stewardship protocol may optimize antifungal prescriptions.^(13)^

Stewardship was used to standardize the institutional supervision of prescribing and monitoring practices for antipsychotics, leading to improved clinical outcomes and a reduction in adverse effects.^(14)^ A critical focus has been on limiting the development of antimicrobial resistance, and efforts to limit the unnecessary use of antimicrobials should be encouraged. The pandemic has allowed for the faster growth of management protocols.^(15)^

We believe that this is the first study to analyze multidrug stewardship in TM encounters related to confirmed or suspected COVID-19 infections. Considering the large number of patients evaluated, even with the rapid expansion of the TM team, extraordinary adherence to current best-practice guidelines was observed.^(3)^ Consequently, in remotely managed patients, we found a much lower rate of antimicrobial prescriptions than that reported in the literature. The population not suspected of having COVID-19 on virtual assessment had multiple differential diagnoses of airway infections. This explains the greater number of and variety (including antibiotics) in medications prescribed to this group.

The limitations of this study include that it was a unicentric retrospective and observational study, and the results may not be generalizable to other locations or health systems.

## CONCLUSION

Guideline training and Telemedicine consultation feedback interventions may lead to lower rates of antibiotic and other antimicrobial prescriptions in suspected and confirmed cases of COVID-19. Multidrug stewardship protocols may improve guideline adherence and reinforce the quality and safety of Telemedicine.
